# Neurocognitive Functions and Behavior in Joubert Syndrome

**DOI:** 10.15844/pedneurbriefs-30-12-3

**Published:** 2016-12

**Authors:** Andrea Poretti, Gwendolyn J. Gerner

**Affiliations:** 1 Section of Pediatric Neuroradiology, Division of Pediatric Radiology, Russell H. Morgan Department of Radiology and Radiological Science, The Johns Hopkins University School of Medicine, Baltimore, MD; 2 Department of Neuropsychology, Kennedy Krieger Institute, Baltimore, MD

**Keywords:** Joubert syndrome, Cognitive functions, Behavior

## Abstract

Investigators from multiple Italian pediatric neurology and neurogenetics departments studied cognitive functions, behavior, and adaptive functioning in large cohort of 54 patients with Joubert syndrome (JS) as part of a prospective, multi-center study.

Investigators from multiple Italian pediatric neurology and neurogenetics departments studied cognitive functions, behavior, and adaptive functioning in large cohort of 54 patients with Joubert syndrome (JS) as part of a prospective, multi-center study. The authors applied standardized, age-appropriate tests to assess development, intelligence, behavior, and adaptive functioning. A global developmental delay was found in 44 (81%) patients. Cognitive functions have been assessed in 49 patients: intelligence (IQ) or general quotient ranged from 15 to 129 (mean 58) and was normal in six (11%) patients. Performance IQ (mean 59) was lower than verbal IQ (mean 67). Scores on subtests of arithmetic and verbal comprehension items were particularly low representing deficits in working memory. A psychiatric diagnosis was reached only in four (7.4%) subjects, but 21 (39%) of 54 patients showed inattention, hyperactivity, social withdrawal, and atypical behaviors affecting daily life. Internalizing problems (anxiety and sociality) were more common than externalizing problems. Adaptive functioning revealed that the motor domain was the area of greatest vulnerability, with a negative impact on personal care, social, and academic skills, while communication skills were relatively preserved. [1]

COMMENTARY. JS is a rare mid-hindbrain malformation that results in the pathognomonic molar tooth sign and hypo-dysplasia of the cerebellar vermis [2]. JS is genetically heterogeneous and is caused by pathogenic variants in more than 30 genes encoding proteins of the primary cilium [3]. Hypotonia, an abnormal neonatal respiratory pattern including apnea and tachypnea, ocular motor apraxia, and ataxia are typical clinical features observed in children with JS. Cognitive impairment and intellectual disability are common in JS and only few patients with JS and normal IQ have been reported so far. The current study adds to the knowledge about cognitive functions in children with JS and shows that cognitive functioning is extremely variable in JS, ranging from severe disability to normal and correlating well with adaptive function. In addition, up to 40% of the children showed behavioral changes, although a psychiatric diagnosis was made only in a few children. Impairment in cognitive functions and behavioral abnormalities in children with JS is in line with the “cerebellar cognitive affective syndrome”, which includes impairments of executive function, deficits in visuospatial skills, linguistic deficiencies, and inappropriate behavior and affect [4]. Knowledge about the type and variability of cognitive functions and behavior in children with JS is important for prognostic and counseling purposes as well as for early initiation of targeted, supportive, rehabilitation therapies to improve functioning and quality of life; however, this may be challenging within the context of the degree of cognitive impairment frequently observed among individuals with JS. As such, use of a combination of standardized neurocognitive measures in conjunction with more experimental methods of examining the developmental trajectories of specific neurocognitive functions will be beneficial. Future studies should also evaluate these outcomes on both types of neurocognitive measures in children with JS, and the relationship with neuroimaging findings and genetic causes.
